# Epitope mapping of an uncertain endogenous antigen implies secretogranin II peptide splicing

**DOI:** 10.12688/f1000research.20633.2

**Published:** 2019-12-05

**Authors:** David R. Howlett, Iain J. Clarke, Russell P. Newton, John E. Hart

**Affiliations:** 1 Wolfson Centre for Age Related Disease, Kings College London, London, SE1 1UL, UK; 2 School of Agriculture and Veterinary Science, Melbourne University, Parkville, Victoria, VIC 3010, Australia; 3 Biochemistry Group, Institute of Life Sciences, Medical School, Swansea University, Swansea, Wales, SA2 8PP, UK; 4 Endocrine Pharmaceuticals Ltd, Tadley, Hampshire, RG26 3TA, UK

**Keywords:** Antibodies, Antigens, Peptides, Epitopes, Bioinformatics, Proteomics, Imaging

## Abstract

**Background**: The search for a tissue-mass reducing reproductive hormone involved a bioassay-guided physicochemical fractionation of sheep blood plasma. This brought forth a candidate protein whose apparent mass on gels and in mass spectrometry (MS) was 7-8 kDa, implying a polypeptide of ~70 residues. Four purification runs gave Edman N-terminal sequences relating to _1_MKPLTGKVKEFNNI _14_. This is bioinformatically obscure and has been resistant to molecular biological investigation. The sequence was synthesized as the peptide EPL001, against which was raised a goat polyclonal antiserum, G530. Used in an antigen capture campaign, G530 pointed to the existence of a novel derivative of secretogranin II (SgII), the neuroendocrine secretory vesicle helper protein and prohormone. The proposed SgII derivative was dubbed SgII-70, yet the sequence commonality between SgII and EPL001 is essentially NNI.

**Methods**: Immunohistochemical (IHC) labelling with G530 is reported within rat, mouse and human cerebrovasculature and in glandular elements of the mouse intestine. Epitope mapping involved IHC peptide preabsorption, allied to deductive bioinformatics and molecular modelling in silico.

**Results**: G530 is deemed monoepitopic in regard to both its synthetic antigen (EPL001) and its putative endogenous antigen (SgII related). The epitope within EPL001 of the anti-EPL001 antibody is inferred to be the contiguous C-terminal _9_KEFNNI _14_. This is so because the G530 blockade data are consistent with the epitope in the mammalian endogenous antigen being part contiguous, part non-contiguous KE·F·NNI, *ex hypothesi*. The observed immunostaining is deduced to be due to pre-SgII-70, which has a non-C-terminal NNI, and SgII-70, which has an N-terminal MLKTGEKPV/N and a C-terminal NNI (these two motifs being in the reverse order in the SgII parent protein).

**Conclusion**: The present data are consistent with the hypothesis that the anti-EPL001 antibody binds to an SgII-related epitope. SgII is apparently subject to peptide splicing, as has been reported for the related chromogranin A.

## Introduction

The deployment of epitope mapping is described here in a factor hunt. Polyclonal antisera (hereafter ‘antibodies’) raised in rabbits and a goat to a synthetic peptide have provided neuroendocrine immunohistochemistry (IHC) images in mammals (human, sheep, rat) and in the embryo of the fruit fly *Drosophila melanogaster* ^1^. But what exactly were the antibodies seeing endogenously? They had been raised as a result of a hunt for a tissue-mass inhibiting reproductive hormone ^2^. A peptide of 7–8 kDa (polyacrylamide gel electrophoresis, MALDI-TOF MS) was found in sheep jugular vein plasma subjected to fractionation via ultrafiltration and guided by assays *in vivo* (internal organ reduction in rats) and *in vitro* (reduced division of rat bone marrow cells), but scant amino acid (aa) sequence data could be obtained before the target molecule was lost to view. In this prior work ^1^ an unambiguous sequence obtained by automated step-wise Edman degradation (Applied Biosystems/PROCISE, Foster City, CA, US) was the N-terminal 14 amino acids MKPLTGKVKEFNNI, synthesized as a peptide designated EPL001. The preceding purification run provided the partially similar ML/KPLTGQAMEF, while a following run delivered the highly similar MKPLT/GKVKxFNNI. On another occasion readings at only four positions could be obtained, providing, however, in-register matches: - - **P** - - - - **V** - - **FN**. This was deemed significant as it involved a maximally purified bioactive fraction derived from ultrafiltered sheep plasma subjected also to gel filtration and anion exchange chromatography. The unambiguous 14-residue sequence is bioinformatically obscure and proved resistant to investigation by molecular biology approaches involving the use of oligonucleotide probes and RT-PCR to find matching sequences in DNA and RNA libraries and the use of anti-EPL001 antibodies to identify cDNA synthesized proteins. Attempts were made to acquire sequence data via digestion with trypsin (porcine), together with MS analysis and interrogation of peptide mass fingerprint databases. No significant hits were seen in a campaign focussed on MALDI-TOF validated bioactive anion exchange fractions from 12 purification runs, three species (sheep, cow, pig), two source materials (ovarian follicular fluid and blood plasma), multiple MS modalities (MALDI-TOF, Delayed Extraction-MALDI-TOF, QTOF, LCQ Deca XP, ESI-QUAD-TOF and LC-MS/MS) and different online search tools such as Mascot and MS-Fit. A carboxypeptidase was deployed on ovine ovarian follicular fluid fractions to achieve C-terminal truncation in a MALDI-TOF MS study, without productive outcome. Factor elusiveness thwarted *de novo* sequencing using MS/MS and also the use of an anti-EPL001 antibody to aid identification by subtraction from spectra.

The 14 residues MKPLTGKVKEFNNI were synthesized as EPL001, as described. A goat anti-EPL001 antibody was raised ^1^ and designated G530. Apart from being used in IHC, G530 was deployed in an antigen capture campaign featuring immunoprecipitation (IP) with liquid chromatography-mass spectroscopy (LC-MS), using two main feedstocks: aqueous extract of rat hypothalamus and fruit fly embryo material ^1^. The former was tested for bioactivity. It proved to have anti-proliferative and pro-apoptotic effects in an assay *in vitro* involving rat bone marrow cells. This inhibitory influence was subject to prior immunodepletion by an anti-EPL001 antibody, except when peptide EPL001 was added as well during the immunodepletion process, achieving preabsorption. Multiple lines of evidence indicated that the mammalian antigen was likely to be a proteoform of secretogranin II (SgII), the neuroendocrine secretory granin helper protein and prohormone. At ~70 residues, this polypeptide derivative was dubbed SgII-70 (pronounced ‘sig two-seventy’). Cryopreserved material in IP/LC-MS delivered no credible candidate. ‘Likely SgII relatedness’ arose from rat hypothalamic aqueous extract subjected to formalin fixation and antigen retrieval. What works in IHC seems to have worked in IP/LC-MS, suggesting factor lability countered. (The target molecule registered weakly in relatively soft MALDI MS but not at all in harsher electrospray ionization.) Meanwhile the fruit fly antigen appeared to be an uncharacterised protein, (UniProt ID Q9W2X8), which was newly recognised as having extensive homology in detail with rSgII ( P10362), making fly Q9W2X8 a probable granin for this and other reasons (e.g. acidic character, multiplicity of dibasic residues, IHC localization etc.) ^1^. Granins both? Coincidence? Identified on the basis of a single tryptic peptide at 5% FDR, Q9W2X8 and rSgII would not normally command attention ^3, 4^, except that each item bears a 5 aa motif from the other’s MS ID peptide, in the same relative position. Another coincidence? The SgII MS ID peptide was _115_IILEALR _121_. The match in the fly protein is _155_ **IILE** SQ **R**_161_(identity 71.4%, similarity 85.7%, no gaps; EMBOSS Needle ^5^). The commonality in regard to the fly’s 23 aa ID peptide is within its first 11 residues, _51_DLQQQRHQQPS _61_. The rSgII match is _64_K **L** R **QQ** A **H** REE **S**_74_ (identity 50.0% across the 10-residue match, similarity 80.0%, no gaps), which is part of its first secretory granule sorting domain (see shortly). Ignoring all other sequence homologies between Q9W2X8 and rSgII, what is the likelihood of two stray proteins of the relevant sizes, 1220 and 619 aa respectively, having the specified matches in any position? The likelihood of any 619 aa protein having a 5 aa match to the fly’s ID peptide is 1 in 5,247. The likelihood of any 1220 aa protein having a 5 aa match to the rat’s ID peptide is 1 in 2,636. The combined theoretical probability of a two-way match occurring by chance is thus 5,247 × 2,636 or ~1 in 14m (see *Underlying data*). The corresponding figures from the UniRef50 database ^6^ of ~24m non-redundant proteins of all sizes are 1 in 6,201 and 1 in 4,378 (personal communication, Chris Mundy, independent bioinformatician, Liverpool, UK, using custom Perl scripts). This yields a combined real-world probability of ~1 in 27m. The two-way 5 aa interrelationship between the rat and fly lead candidates is therefore highly unlikely to be due to chance ^7^.

The G530 IP/LC-MS campaign yielded a protein identified by the MS software as Q8CGL8, a splice variant of rSgII of 37.1 kDa, having 322 residues. EPL001 has successfully preabsorbed a band of this size in a western1, as well as one at 7–8 kDa. Q8CGL8 was the only item snared in both of the two forms of antigen retrieval used and so was to that extent the sole mammalian candidate, but SgII-70 itself was not bagged. In regard to EPL001, preliminary mapping ^1^ of the epitope – defined as that part of an antigen molecule to which an antibody binds – involved dot blot analysis with G530 of three peptides: full length EPL001 and two component peptides, the N-terminal _1_MKPLTG _6_ and the C-terminal _7_KVKEFNNI _14_. This showed that the synthetic epitope (singular for parsimony) resides in the C-terminal section of EPL001. Mammalian Q8CGL8 has the EPL001 C-terminal match V---NNI, while fly Q9W2X8 has K----NNI, sketchy resemblances both. Is the EPL001 sequence really related to these proteins?

A chance observation provided a platform for the current investigation, which amounts to an attempt to get beyond the frustrating vagaries of purification and instead use G530 in IHC to elucidate the primary sequence of whatever it is that the antibody sees endogenously, putatively SgII-70. SgII has a pair of domains which sort the protein intracellularly into secretory vesicles ^8^. It was noticed that the ovine-derived EPL001 sequence, MKPLTGKVKEFNNI, finds a nine-residue resemblance in the second sorting domain of sheep SgII ( W5QEU8), a homologue of which exists in fly Q9W2X8. The nine-residue string, ‘sSgII-9’, is _367_ MLK TGE KPV _375_ (residue numbering with signal sequence). The shaded residues match those from the front half of EPL001, in the form of three doubletons, separated by singleton matches to the residues in the second half of EPL001. One of the Edman sequences reported here actually starts ML/K. Disregarding the apparent non-random interleaving of the front and back halves of EPL001, Spearman’s rank correlation between sSgII-9 and EPL001 is 0.59, a moderate positive correlation. The probability of a nine-residue partial anagram of EPL001’s 11 residues (i.e. minus NNI) occurring by chance in a typical ovine protein is about 1 in 146,000, as previously calculated ^1^. Going further, EPL001 and sSgII-9 have the same initial residue: methionine. The probability of this is 1 in 11. The overall likelihood therefore of there being a methionine-commencing nine-residue anagram is 1/146000 x 1/11 or about 1 in 1.6m. The likelihood of there being a nine-residue anagram and NNI in the same protein is tinier still. (NNI probably has sorting domain relatedness too ^1^, like sSgII-9.) Why EPL001 might be an encoded version of sSgII will be considered later (see *Discussion*). Comparing EPL001’s C-terminal section with sSgII sequence elements sets up the prediction that the endogenous epitope could involve six residues, thus:

$${\;}_{367}\text{ML}\underline{\text{K}}\text{TG}\underline{\text{E}}{\text{KPV}}_{375}{\;}_{\text{?}}\text{K}{\;}_{?}\underline{\text{F}}{\;}_{236}{\underline{\text{NN}\text{I}}}_{238}$$

Or thus, reading sSgII-9 in reverse:

$${\;}_{375}\text{VP}\underline{\text{KE}}{\text{GTKLM}}_{367}{\;}_{\text{?}}\text{K}{\;}_{?}\underline{\text{F}}{\;}_{236}{\underline{\text{NNI}}}_{238}$$

These possibilities can be represented as K·E·F·NNI and KE·F·NNI, respectively. There are numerous other combinations of three or more residues from these sSgIIs sequence elements that match the order of residues in EPL001’s C-terminal section, such as K·V·F·NNI and V·KE·F·NNI. All are mixed, i.e. part contiguous, part non-contiguous, except NNI.

This paper attempts to deduce the endogenous epitope of the G530 anti-EPL001 goat antibody in mammals, via IHC peptide preabsorption studies on selected tissues (cerebrovasculature, gut), aided by deductive bioinformatics and molecular modelling *in silico*. Preabsorption, i.e. mixing of the antibody with antigen prior to application of the antibody, to block staining, has been achieved in western blotting with the C-terminal EPL001 peptide but not with the N-terminal peptide, in regard to aqueous extract of rat hypothalamus purified using an immunoaffinity column ^1^. Both the western blotting and the immunopurification used G530. IHC is not described in a review of epitope mapping methods ^9^, but is comparable to ELISA-based peptide-panel techniques for dissecting antigen-antibody interactions. IHC was adopted here particularly in the face of target molecule recalcitrance to MS analysis.

The hypothesis here is that the G530 anti-EPL001 antibody binds to a SgII-related epitope; the null hypothesis is that it binds to something else. The hypothesis informed preabsorption peptide design and predicts that the endogenous epitope is probably a part non-contiguous version of EPL001’s presumed contiguous epitope. Data consistent with the SgII hypothesis and its epitope prediction are presented herein. This first attempt to elucidate the primary structure implies that SgII-70 is the product of peptide splicing.

## Methods

### Antibody

The goat anti-EPL001 antibody was chosen for this IHC investigation because it had been used with success in the antigen capture campaign ^1^ to disclose the target molecule’s apparent relatedness to SgII. Prior published IHC images have, however, been obtained predominantly using rabbit antisera, preferred in this application. The goat polyclonal antiserum (G530) was raised as described elsewhere ^1, 10^. A cysteine EPL001 peptide was synthesized conjugated at its N-terminus with the carrier protein KLH. The goat was injected simultaneously with antigen (400 μg) in PBS mixed with an equal volume of complete Freund’s adjuvant followed by eight booster injections at monthly intervals. An antibody dilution curve was obtained ^1^. Titre was also established via IHC, with blockade of rat and ovine hypothalamic staining by EPL001 at 0.5 mg/ml. This and other examples of IHC preabsorption have been described previously ^1^. No staining was seen with pre-immune serum. An antibody to LRP1 (ABP-PAB-10774) was obtained from a commercial supplier (Allele Biotech, San Diego CA), as was an antibody to SgII (ab192824, rabbit polyclonal to chromogranin C/SgII, raised to a recombinant fragment within human chromogranin C/SgIIaa 1–277; Abcam UK).

### Peptides

Peptides for use in IHC competition studies were synthesized by a commercial supplier (Peptide Protein Research Ltd, Fareham, UK). The peptides were manufactured to GLP using Fmoc solid phase synthesis. Purification involved RP-HPLC using water and acetonitrile as the mobile phases. Peptides were then analysed via LC-MS to determine mass and purity. All peptides were stored at -20°C prior to use. Amino acid sequences are given in Table 1, together with notes on provenance. A control peptide was deployed, in the form of EPL030. This is a random scrambling of the amino acid sequence of EPL001. Peptide design was informed by an earlier analysis ^1^, expanded upon in *Introduction*, which conjectures that the EPL001 sequence can be decoded to reveal SgII.

**Table 1.** Peptide panel. Amino acid sequences, proprietary codes, species background, relationship to EPL001 or SgII and ability to preabsorb in IHC the anti-EPL001 antibody G530. Peptides were used at 10 μg/ml (vs G530 at ~1 μg/ml).

Peptide	Species	Description	Blockade of G530 labelling (gut & cerebrovascular)
MKPLTGKVKEF **NNI** (EPL001)	Sheep ( *Ovis* *aries*)	Edman N terminus of candidate polypeptide for inhibitory hormone, obtained via bioassay guided fractionation	Yes
DEDDVYKT **NNI** AYEDVVGGE	Rat ( *Rattus* *norvegicus*)	‘Secretogranin II relatedness’ arose from G530 purified rat hypothalamus: section of rSgII ( P10362) bearing EPL001’s **NNI**, which is also part of the EM66 processed SgII peptide. SgII in sheep (W5QEU8) & human ( P13521) are DEDDIYKA **NNI** AYEDVVGGE	No
KRSKEQKK **NNI** SHHNYKLKN	Fruit fly ( *Drosophila* *melanogaster)*	Section of G530 purified fly protein (Q9W2X8) bearing EPL001’s **NNI**	No
MLKTGEKPVKF **NNI** KGLEQF (EPL122)	Sheep	Speculative splicing of sections of sSgII	No
MLKTGEKPVKF **NNI** (EPL142)	Sheep	Second sorting domain of sSgII spliced to **NNI**; anagram of EPL001	Yes
MLKTGEKPVFK **NNI** (EPL143)	Sheep	Ditto with KF reversed	Yes
MKPVF **NNI** (EPL801)	Sheep	Shortened version of EPL142	Yes
MLKTGEKPN (EPL373)		Second sorting domain, hSgII-9	No
MKPVFN (EPL601)	Sheep	Shortened version of EPL801	No
KLKMNGKNIEPVFT (EPL030)	Sheep	Sequence of EPL001 randomly scrambled as control peptide	No
KEF **NNI** (EPL536)	Sheep	C terminus of EPL001	Yes
EF **NNI** (EPL545)	Sheep	Ditto	Yes
GKV, KVK, VKE, KEF, EFN, FNN, **NNI**	Sheep	Triplets from the epitope-relevant C-terminal section of EPL001	Only **NNI**
F **NNI**, FNNA, FNAI, FANI, A **NNI**	Sheep	C-terminal tetramer of EPL001 and alanine substituted variants thereof	Yes, but A **NNI** at the highest concentration only ( Figure 5)

### Immunohistochemistry

Brain, small intestine, kidney and spleen tissue was obtained from mice (six C57/Bl6 male mice, supplied by Charles River Laboratories, approximately six months old and weight 35–45g). Mice were housed with free access to Global Rodent Maintained Diet (Harlan Teklad) and water. They were maintained in an ambient temperature of 21±1°C under a controlled light–dark photocycle (12:12 h), with lights on at 07:00 h. Mice were humanely euthanised by overdose of sodium pentobarbitone). Brain tissue was also obtained from rats and humans. The rat details are as follows: four male Wistars supplied by Charles River Laboratories, six months old, 125–150 g; housed under a 12h light/dark cycle with *ad libitum* diet (Global Maintained Diet, Harlan Teklad); euthanasia via a sodium pentobarbitone overdose. The human details were thus: post-mortem cortex, aged control subjects, two male and two female, age 76–87 years; see *Ethics*. Formalin-fixed, wax-embedded blocks, cut into 7-μm sections and mounted onto slides, were used for IHC. Mounted sections of cerebral cortex from bovine brain were obtained from AMSBIO Biotechnology, Abingdon, OX14 4SE, UK. Sections were dewaxed and rehydrated using Histoclear and alcohol dilutions. Antigen retrieval was carried out by microwaving the sections for ten minutes in citrate buffer pH 6.0. Following blocking of endogenous peroxidases (0.3% H _2_O _2_ in PBS for 30 minutes, for DAB sections only), sections were incubated overnight with primary antibody at a dilution of 1:4000. In initial preabsorption experiments, G530 was preincubated with a ten-fold excess of competing peptide for 30 minutes, before being added to the sections. Peptides were used at 10 μg/ml (vs G530 at ~1 μg/ml). Peptides were initially dissolved at 10mg/ml by the addition of 10μl of 10% acetic acid to 1mg of peptide, followed by further addition of deionised water to give a final concentration of peptide of 10mg/ml. Aliquots of 10ul of each peptide were kept frozen at -28°C for future use. In typical preabsorption experiments, antibody G530 was incubated for 30 minutes at a concentration of 1μg/ml (protein concentration) in the presence or absence of competing peptide at 10μg/ml. Dilutions of G530 and peptides were made in primary incubation buffer (PBS + 0.3% Triton X-100 + 2% bovine serum albumin). When no competing peptides were present, a ‘blank’ was incubated with the G530, the blank comprising 10μl of 10% acetic acid plus 90μl of deionised water with subsequent dilution of this in primary incubation buffer to the equivalent of the 10μg/ml peptide concentration. Development of the sections was performed using biotinylated secondary antibodies at a 1:500 dilution (BA1000/RRID AB_2313606; BA-2000/RRID AB_2313581; BA5000/RRID AB_2336126), ABC reagents (PK-6100/ RRID AB_2336819, used according to manufacturer’s instructions) and a DAB kit (all Vector Laboratories, Peterborough, UK). Sections were briefly counterstained with Mayer’s hematoxylin solution before dehydration, mounting with DPX and coverslipping. For control experiments, the secondary biotinylated antibody was omitted. In some experiments, following incubation with G530 (± competing peptide), the secondary antibody was anti-goat Alex Fluor 568 used at a 1:500 dilution (A11079/AB_2534123; Invitrogen, ThermoFisher Scientific, Waltham, MA, USA).

G530 was preabsorbed with tripeptides based on the epitope-relevant C-terminal section of EPL001. This was with and without the EPL001 parent peptide. The aim of the ‘with EPL001’ protocol was to block EPL001’s previously demonstrated inhibition of IHC staining.

In an IHC dose-response study involving the EPL001 C-terminal tetrapeptide FNNI and alanine substituted versions thereof the tissue consisted of serial sections of mouse small intestine and coronal sections of rat brain. The G530 antibody (final dilution 1:4000) was incubated in primary incubation buffer (PBS + 0.3% Triton X-100 + 2% bovine serum albumin) with peptide (dissolved in high purity water) at dilutions between 0.1 ng/ml and 1 μg/ml (final peptide concentration) for 30 min prior to addition to sections (tissue dewaxed and rehydrated; antigen retrieval with citrate at pH 6.0). Sections were incubated overnight at 4°C. Further development was with the anti-goat secondary antibody Alexa Fluor 568. Serial images of matching features, either within the walls of small cerebral blood vessels or in glandular elements of the mouse small intestine, were analysed using ImageJ version 1.52i with a fixed threshold to give a value for ‘area labelled’.

### Molecular modelling

Models *in silico* were developed using Molecular Modelling Pro Plus, version 6.22, and ChemSite, version 5.10, produced by ChemSW (Accelrys Inc., San Diego, USA; Avogadro is an open-access alternative). Models were constructed by sequential additions of amino acid residues. Each model was adjusted in conformation to minimize energy levels: energy minimization was carried out in 1-fs time steps, to a total of 10,000 fs, with 100 equilibrium steps per iteration. Iterations were continued until six repeat iterations yielded no change in energy gradient. Analysis of interatomic distances mostly involved atoms in amino acid side-chains. Distances between pairs of atoms were computed automatically after atoms were selected manually on-screen. Each measurement was repeated twice more after closing the model and reloading to verify the initial measurement. For EPL001’s _9_KEFNNI _14_, nine atoms were selected from side chains and two from the peptide backbone ( Figure 6). This permitted 46 measurements, each atom to every other atom: a-b, a-c etc. Distances between the same atoms were calculated for KEFNNI as a free peptide, with comparisons reducing in number for free EFNNI through free FNNI to free NNI. Other ad hoc interatomic measurements are described in *Results*.

### Statistical analysis

Calculations to provide *p* values in the IHC image analysis were conducted using unpaired *t*-tests. A chi-squared test was used for interatomic distance comparisons in the molecular modelling, with measurements from the EPL001 model *in silico* representing expected (E) interatomic distances and measurements from the modelled free peptides (KEFNNI, EFNNI, FNNI, NNI) as observed (O) distances. Chi-squared values were calculated on the basis of (O-E) ^2^/E.

### Ethics

All experimental procedures were conducted in strict compliance with applicable laws, regulations, rules and professional standards, with appropriate ethical oversight. The G530 antiserum was raised in compliance with the Australian Prevention of Cruelty to Animals Act 1986, with procedures approved by the relevant Animal Ethics Committee. The provision of animal tissue for histology in the UK was licensed in accordance with the Animals (Scientific Procedures) Act 1986. Human post-mortem tissue sections were provided, with ethical approval, by courtesy of Brains for Dementia Research Network (Alzheimer’s Society and Alzheimer’s Research UK). Data integrity has been maintained throughout, without outlier exclusions and with appropriate recording and archiving.

## Results

### Labelling of antibody G530

**Figure 1.**
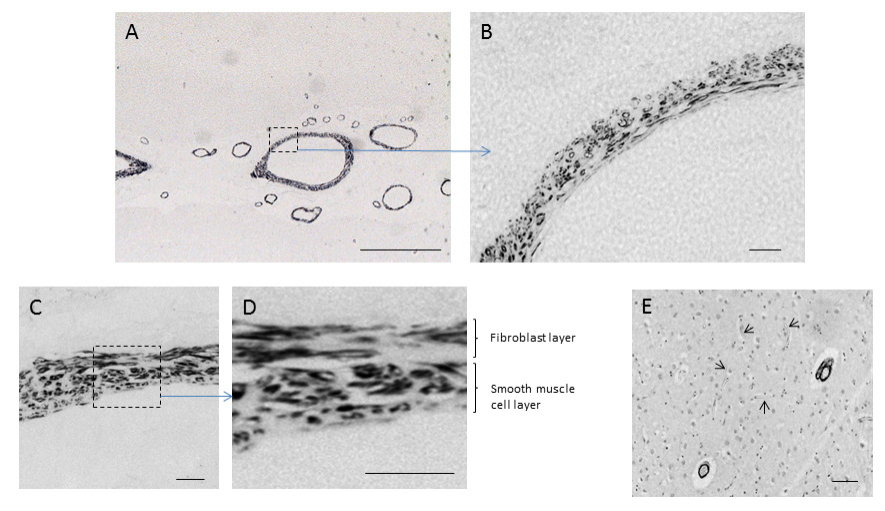
Immunohistochemical labelling within the walls of human cerebral blood vessels. Sections of human cingulate cortex were labelled by antibody G530 as described in *Methods*. ( **A**– **D**) Cerebrovascular wall labelling at differing magnifications (scale bars: **A** = 500 μm; **B**, **C** = 25 μm; **D** = 10 μm). ( **E**) Lack of labelling (arrowed) in cerebral capillary walls (scale bar = 500 μm).

**Figure 2.**
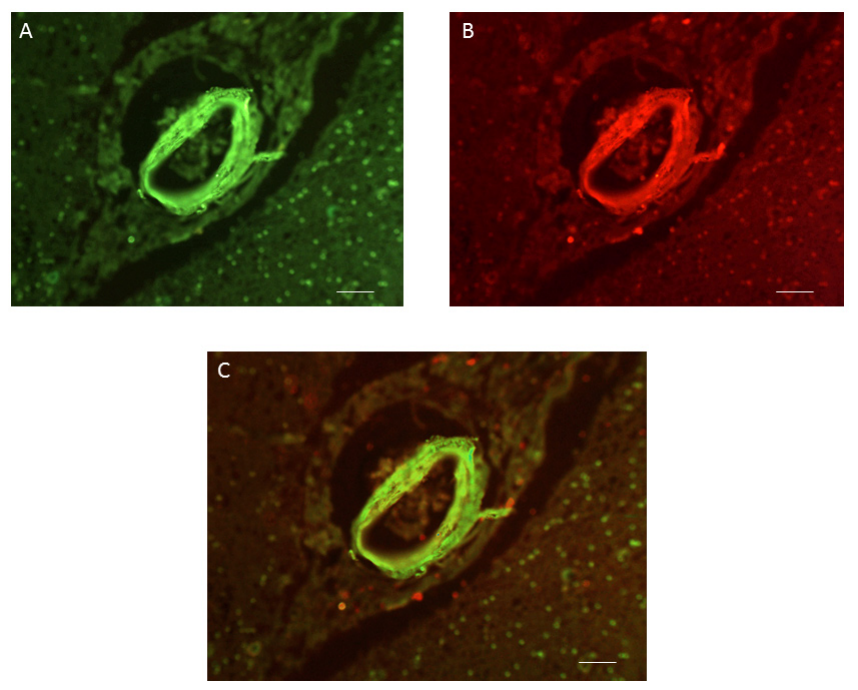
Immunofluorescent labelling within the walls of human cerebral blood vessels. Sections of human cingulate cortex were labelled by antibody G530 as described in *Methods*. ( **A**) Labelling by an antibody to LRP-1. ( **B**) Labelling by G530. ( **C**) Merge of ( **A**) and ( **B**). Scale bars, 50 μm.

**Figure 3.**
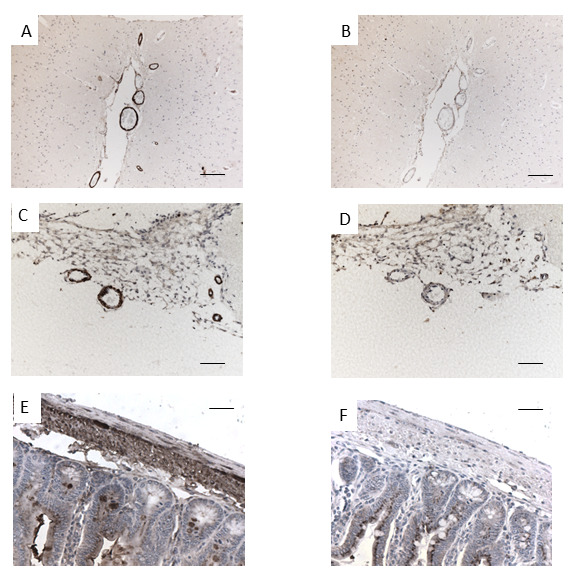
Preabsorption of G530 labelling by peptide EPL001. Wax sections of cerebral cortex, human ( **A**, **B**) and mouse ( **C**, **D**), and of mouse small intestine ( **E**, **F**) were incubated with either antibody G530 ( **A**, **C**, **E**) or G530 preabsorbed by a ten-fold excess of cognate peptide EPL001. Scale bars: ( **A**– **D**) = 100 μm; ( **E**, **F**) = 25 μm.

Antibody G530, raised to the 14mer peptide EPL001, demonstrated labelling within the walls of cerebral blood vessels in mouse, rat and human, in a manner not previously described. The labelling was observed in the walls of arteries and arterioles, but not capillaries and appeared to be associated with the fibroblast and smooth muscle layers surrounding the contractile vessels ( Figure 1). Co-labelling employing an antibody to LRP1 confirmed the vascular G530 labelling to be within blood vessel walls ( Figure 2). In mouse small intestine sections, G530 produced labelling within the muscularis mucosae and possibly some columnar epithelium but no labelling within lamina propria ( Figure 3), consistent with prior findings ^1^. In other tissues evaluated prior to the study proper, labelling of blood vessel walls was observed within mouse spleen and kidney and bovine (this paper) and ovine brain ^1^. Labelling was noted within cortical neurons of the species examined, with preabsorption in one series by EPL001 but not in another. The greater reliability of the cerebrovascular staining commended this as a focus in the present study. An antibody to SgII did not produce any labelling of mouse, rat and human cerebral blood vessels; although labelling was observed in the mouse small intestine, this bore no relation to G530 labelling. Raw images used to generate Figure 1– Figure 5 and Table 1 are available as *Underlying data* ^11– 19^.

**Figure 4.**
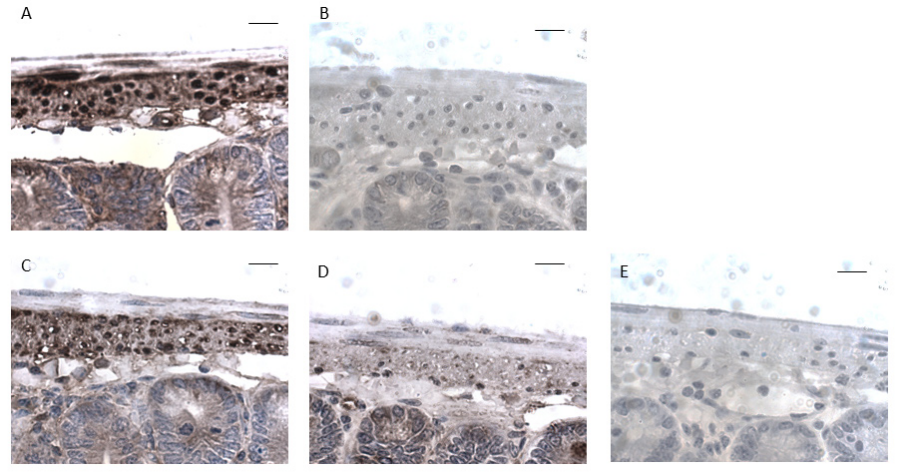
Preabsorption of G530 labelling of elements within the wall of mouse small intestine by different peptides. G530 was preincubated for 30 min with a x10 excess of potential preabsorption peptide before application to wax sections of mouse small intestine. ( **A**) G530 alone. ( **B**) G530 + EPL001. ( **C**) G530 + EPL030. ( **D**) G530 + EPL142. ( **E**) G530 + EPL143. Amino acid sequences of peptides are shown in Table 1. Scale bars, 10 μm.

**Figure 5.**
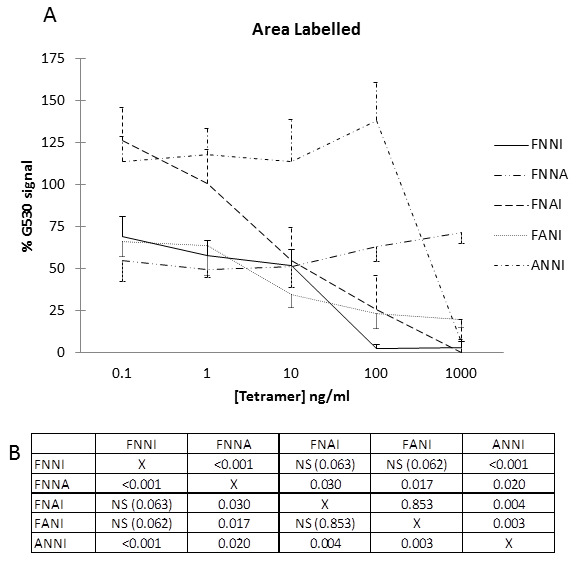
Preabsorption of G530 labelling by C-terminal tetramer (FNNI) and alanine substituted variants thereof. ( **A**) G530 (dilution 1:4000) was incubated for 30 min with tetramers at concentrations of 0.1 to 1000 ng/ml before application to the sections for IHC. Graph shows staining intensity (ImageJ) expressed as a percentage of G530 signal in absence of tetramer. Consistency of results invited data aggregation (murine gut, rat cerebrovasculature). Data are expressed as mean ± SEM of four determinations. The horizontal axis effectively represents the full preabsorption achieved with EPL001 at all concentrations. ( **B**) Statistical comparisons ( *p* values) are shown in the matrix for data points at 100 ng/ml (~0.2 μM).

**Figure 6.**
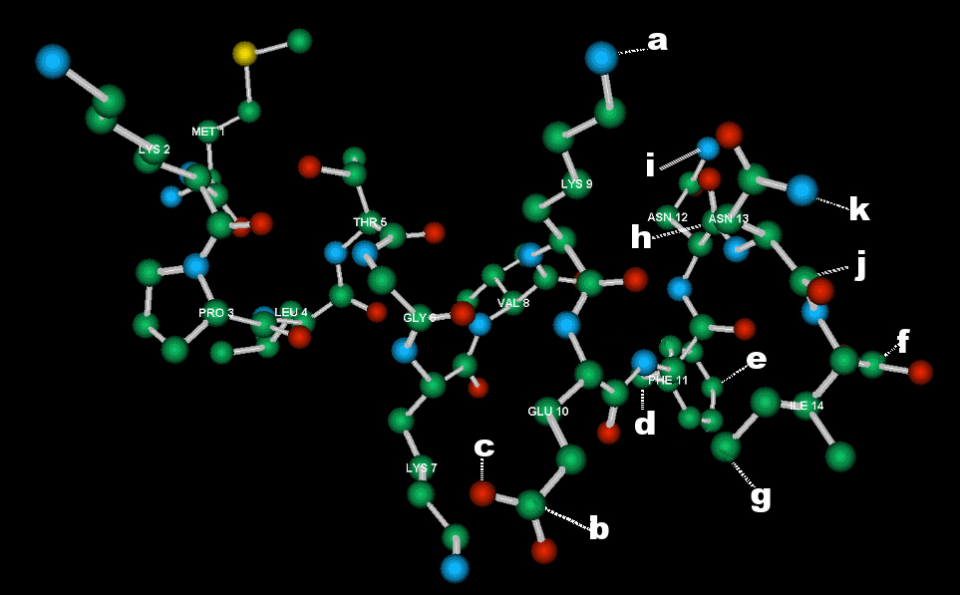
Model *in silico* of peptide EPL001. Minimized molecular model of the 14mer MKPLTGKVKEFNNI. Carbon = green; nitrogen = blue; oxygen = red; sulphur = yellow. Hydrogen atoms omitted for clarity. Within the putative epitope KEFNNI (LYS 9 – ILE 14) atoms are indicated (a–k) that were used to determine interatomic distances. a = nitrogen in the side-chain of K9; b = delta carbon in E; c = hydroxyl oxygen in the side-chain of E; d = beta carbon in F; e = gamma carbon of F; f = peptide bond carbon of I; g = delta carbon of I; h = gamma carbon of N13; i = nitrogen of the side-chain of N13; j = peptide bond carbon of N12, k = nitrogen of side-chain of N12.

### Peptide competition experiments

In initial peptide competition experiments, labelling within both human and mouse cerebral blood vessels and mouse small intestine was prevented by preincubating G530 with its cognate peptide EPL001 ( Figure 3). Table 1 shows that peptides with a C-terminal NNI block labelling. Thus, effective blockers, in straightforward competition with the native antigen for antibody binding, are the NNI-concluding 14mers EPL142 and EPL143 ( Figure 4), MKPVFNNI and the EPL001 C-terminal fragments KEFNNI, EFNNI and FNNI. Ineffective are six peptides: the fly and rodent SgII 20mer homologs and the 20mer extended form of EPL142 (EPL122), all with mid-sequence NNIs; a scrambled-sequence version of EPL001 lacking NNI but with a mid-sequence NI, KLKMNGKNIEPVFT; and two peptides lacking NNI altogether but terminating in N, in one case FN: MLKTGEKPN and MKPVFN. NNI was the only one of seven EPL001-related trimers that preabsorbed G530. Given this result, the attempt was not made to co-administer NNI and EPL001, inhibitors both. The other six trimers co-administered separately with EPL001 were ineffective as counter-inhibitors. EPL001 administered alone achieved near-total preabsorption across a range of concentrations (mean ± SEM, n = 4): 0.1 ng/ml = 0.56 ± 0.56; 1 ng/ml = 0.30 ± 0.30; 10 ng/ml = 0 ± 0; 100 ng/ml = 0.09 ± 0.05; and 1 μg/ml = 0 ± 0. Preabsorption titration across the same range of concentrations then focussed on FNNI, EPL001’s C-terminal tetramer, via alanine substitutions. Three tetramers in the form F - - I returned concentration-response curves; two tetramers in the form - - - I and F - - - did not ( Figure 5). Labelling was actually increased by ANNI, except at the highest concentration. Preabsorption with EPL001’s C-terminal tetramer FNNI was asymptotic at the highest concentrations, with 2.7% staining (G530 alone bring 100%) at 100 ng/ml (~0.2 μM/L) and 3.5% at 1 μg/ml. Preabsorption was further investigated using EPL001’s C-terminal hexamer KEFNNI. At 100 ng/ml (~0.13 μM/L) the area of staining (as % of G530 staining without competing peptide) was 29.53 ± 9.69 (n = 8, *p* = 0.062, not significant (NS) vs preabsorption by EPL001 at 100 ng/ml), while at 10 μg/ml it was 4.7 ± 2.4 (n = 3, *p* = 0.12, NS). (The corresponding figures for MKPVFNNI were 44.40 ± 11.85, n = 8, *p* = 0.028 vs EPL001 and 3.92 ± 1.97, with n = 3, *p* = 0.11, NS.) In approximate terms, the IC _50_ preabsorption trend is as follows: EPL001 < 0.1 ng/ml; FNNI ≤ 10 ng/ml; KEFNNI ≤100 ng/ml.

### Interatomic distances between residues of different peptide sequences

The interatomic distances calculated via molecular modelling ( Figure 6) yielded on statistical analysis a proportion of (O-E) ^2^ values >10, betokening a big difference between observed and expected, as follows: KEFNNI, 39%; EFNNI, 11%; FNNI, 5%; NNI, 25%. Free FNNI is indistinguishable from EPL001’s _11_FNNI _14_ in statistical terms (Chi ^2^ = 4.54, degrees of freedom (dof) = 19, 99.5% confidence level). The null hypothesis, that there is no correlation between EPL001 and, separately, KEFNNI (Chi ^2^ = 111.31, dof = 45, NS), EFNNI (Chi ^2^ = 23.67, dof = 35, NS) or NNI (Chi ^2^ = 4.36, dof = 7, NS), was accepted for the other comparisons of interatomic distances (see *Underlying data*) ^15^. Interatomic distances were also compared between the Ks and E in the C-terminal section of EPL001 ( _7_ **K** V _9_ **K**_10_ **E** FNNI) and those in sSgII-9: ML _3_ **K** TG _6_ **E**_7_ **K** PV (see *Introduction*). The same side chain measurements (in Å) were made for these sequences as for the K and E in EPL001’s KEFNNI. Using the letters given in Figure 6, EPL001’s **K7-E10**: a-b = 3.06, a-c = 3.49. EPL001’s **K9-E10**: a-b = 11.09, a-c = 12.24. sSgII-9’s **K3-E6**: a-b = 3.12, a-c = 2.77. sSgII-9’s **E6-K7**: a-b = 11.58, a-c = 11.92. Interatomic measurements (20 in total) were made of the FNNI in an *in silico* model of MKPVFNNI (EPL801, Table 1). These were compared with FNNI measurements in EPL001’s _9_KEFNNI _14_ and in free KEFNNI. The FNNI distances for EPL001 and EPL801 are similar, with the KEFNNI results very dissimilar (see *Underlying data*) ^15^. F-I measurements for EPL001 are as follows (using letters as given in Figure 6), with EPL801 data in parentheses: d-f = 6.79 (6.34), d-g = 5.49 (7.40), e-f = 7.45 (6.90), e-g = 8.45 (8.96). The figures for KEFNNI are: d-f = 10.57, d-g = 13.56, e-f = 12.21, e-g = 14.01. A chi-squared test of all 20 measurements with EPL001 data as expected showed that the FNNI in MKPVFNNI is highly similar to _11_FNNI _14 _in EPL001: Chi ^2^ = 4.33, dof = 19, 99.5% confidence level. The K2-F5 gaps in MKPVFNNI are a-d = 8.91 and a-e = 9.42, taking ‘a’ as the side-chain nitrogen of K2. For comparison, KEFNNI’s figures for K1-F3 are a-d = 8.69 and a-e = 11.14, while EPL001’s K9-F11 gaps are a-d = 11.38 and a-e = 10.37.

## Discussion

This report describes the unusual situation where the identity is unclear of an endogenous antigen of an antibody raised to a synthetic peptide, itself of problematic sequence. Epitope mapping is being used here to help solve a purification puzzle, the pieces of which are ‘EPL001’, ‘G530’ and ‘SgII’. It has been demonstrated previously ^1^ that a goat polyclonal antiserum (G530) raised to the synthetic peptide MKPLTGKVKEFNNI (EPL001) labels neuroendocrine and other tissues in various mammalian species and that the endogenous antigen likely relates to secretogranin II (SgII). To determine the endogenous epitope at amino acid resolution, the present report uses IHC, after previous immunoblotting showed that the synthetic epitope resides within or comprises KVKEFNNI, EPL001’s C-terminal section ^1^. Conventional epitope mapping techniques involve X-ray crystallography, nuclear magnetic resonance spectrometry, MS, phage display, ELISA and mutagenesis ^3^, with electron cryomicroscopy a recently developed method of revealing the structures of antibody-antigen complexes. MS-based epitope mapping has been reviewed ^20^, with studies involving synthetic peptide antigens ranging from 47 residues down to 14, as here. In the latter case of a 14mer peptide ^21^, antibodies were interrogated via a panel of synthetic peptides, with alanine substitution, using immunoaffinity-MS and, separately, dot blotting and ELISA. Epitope mapping has been reported for a granin, chromogranin A, using ELISA with a panel of overlapping peptides ^22^. The approach used in the present study has been to probe an enigmatic native antigen *in situ* aided by formalin crosslinking and antigen retrieval, because of its resistance to purification and MS analysis and its apparent lability. A panel of IHC preabsorption peptides included overlapping trimers, plus a series of alanine-substituted C-terminal tetramers. Immunolabelling is described within the walls of mouse, rat and human cerebral blood vessels and in the wall of the mouse small intestine. Although the EPL001 peptide displays anti-proliferative and pro-apoptotic activities *in vitro* ^10^ and tissue-mass reducing properties *in vivo* ^23^ (with relevant immunoneutralizations by anti-EPL001 antibodies *in vitro* ^1, 10^ and *in vivo* ^23^), implying that an endogenous analogue might do likewise, functional aspects are not a concern in the current report. Neither is the import of the histomorphology. Instead, for antigen elucidation, the focus is exclusively on what the anti-EPL001 antibody binds endogenously in a detailed molecular sense.

The efficacy of preabsorption and the absence of non-specific binding confirm the ostensible specificity of G530. But to what is it specific? The deployment of two-dozen 3–20mer synthetic peptides in competitive preabsorption studies has demonstrated the importance of a C-terminal NNI in blocking G530 labelling. Although a phenylalanine residue adjacent to the NNI sequence does not appear essential for competition, as the NNI trimer preabsorbs G530 on its own, three N-containing tetramers in the form F - - I, including EPL001’s C-terminal FNNI itself, each delivered the semblance of a concentration-response curve, while two tetramers in the forms - - - I and F - - - did not. In a preliminary analysis, the epitope in the mammalian endogenous antigen could be a C-terminal tetramer, FNNI. Granted EPL001’s ovine provenance, there are no full-length proteins with a C-terminal FNNI in that part of the TrEMBL database ^24, 25^ devoted to *Ovis aries* (personal communication, Chris Mundy, independent bioinformatician, Liverpool, UK, using custom Perl scripts). Of the 26,443 ovine predicted proteins 96% can be discounted by considering only those items containing NNI. This is the ‘NNI-ome’ (see *Underlying data* ^26^). Comprising 1,100 predicted proteins, the ovine NNI-ome boasts 1,181 NNIs in all. The assumption here is that one of these motifs relates uniquely to the NNI in EPL001’s sequence MKPLTGKVKEF **NNI**. Among the ovine NNIs are 42 FNNIs, 7 of which are in the form EFNNI. None display EPL001’s KEFNNI and none otherwise connotes EPL001. Three NNIs are C-terminal ( W5Q2R9, W5QFS6 and C5IS99) but EPL001 is otherwise not evoked by these sequences and at 152/153 residues these items are anyway overlarge, the sought-for factor being of ~70 residues (see *Introduction*). The NNI-ome includes 17 predicted proteins of less than 100 residues. Beyond possessing NNI (in two cases in the form FNNI), none of these resembles EPL001. A sift of the NNI-ome can be achieved on the assumption that the initial methionine of EPL001 is a correct reading. There are 25 sequences of 14 residues in the form MxxxxxxxxxxNNI, with one having FNNI ( W5Q754). Beyond having additional stray single-residue correspondences, these are all unlike EPL001. The Method of Exclusion can be used to eliminate all 25 from consideration in fact. This involves excluding all candidate Mxxxxxxxxxx sequences bearing non-EPL001 residues, i.e. ARNDCQHISWY, plus M, as that is used up as the first residue. Within the NNI-ome’s 1,100 proteins there are 22,207 methionine residues, discounting signal sequence initiator Ms. This is the pool of candidates for the M in EPL001. Shorn of NNI, EPL001 has 9 unique residues: MKPLTGVEF. Of Mxxxxxxxxxx sequences in the ovine NNI-ome 38 are composed exclusively of these 9 residues, counting repeat aa as one. None has all 9. Five have 8, the rest having from 7 down to three. The five with 8 EPL001-less-NNI residues are as follows, described by similarity with other mammalian proteins: W5Q754 (titin, structural, MLKKTPVLKKG); W5PWS5 (dynein, structural, MLFVGPTGTGK); W5PP00 (ubinuclein-2, nuclear, MPKVVPTLPEG); W5PWP9 (hormonally up-regulated neu tumour-associated kinase, enzyme, MLTGTLPFTVE) and W5QEU8 (secretogranin II, secretory vesicle, MLKTGEKPVEP). The stand-out candidate from amongst these five is the last, SgII, an established prohormone of interest from the purification campaign, having commonalities with EPL001 beyond residue complement (see Introduction). The match is to SgII’s second sorting domain. Thus, bioinformatics of a serendipitous kind, and now more systematically, point to the same part of the same protein. The model here, on the basis of NNI-omics, is that EPL001 is a reading from non-contiguous parts of a protein’s sequence, the NNI reading being sequential, the Mxxxxxxxxxx reading after M not. This model will be expanded upon later, in terms of proposed SgII peptide splicing and sequencing considerations.

Among endogenous antigens, 10% are contiguous in that they involve a sequence of neighbouring amino acids along a protein backbone ^20^. The other 90% are either entirely non-contiguous, comprising non-consecutive amino acids brought together in space by protein folding, or mixed contiguous and non-contiguous. Antibodies that recognize non-contiguous epitopes can nonetheless cross-react with the contiguous aa in short synthetic peptides, enabling the antibody binding site to be determined ^27^ It has been hypothesized that the native antigen of G530 is related to SgII and that it is mixed, perhaps K·E·F·NNI or KE·F·NNI, corresponding to EPL001’s conjectured maximum likely contiguous epitope _9_KEFNNI _14_ (see *Introduction*). If KEFNNI endogenously were fully contiguous, then the triplets KEF, EFN and FNN might be expected to block the antibody, but they don’t – though FNN extended to FNNA does to a modest extent ( Figure 5). That NNI alone among the trimers successfully preabsorbed G530 indicates that NNI is probably the only contiguous epitopic element endogenously. The sole NNI in the SgII parent protein is not preceded by an F (see Table 1, third item, for the relevant sequences of rSgII, hSgII and sSgII). This implies that the endogenous epitope is thus at least minimally mixed, in the form of F·NNI – and that this is why alanine substitution can be used successfully to probe this part of the epitope. Epitopes cannot be predicted reliably from amino acid sequences, according to a survey of MS epitope mapping, with structure-based rules lacking ^20^. This review found 57 relevant papers from 1986–2015, disclosing 63 epitopes. These ranged in size from 4–71 amino acid residues, with a mean of 15, median of 12 and mode of 8. The present epitope might be F·NNI, but smallness renders this unlikely. That free FNNI is markedly less preabsorptive than EPL001 supports the view that there is more to the epitope than F·NNI.

‘SgII relatedness’ arose on the basis of the present antibody’s deployment in an antigen-capture campaign directed at rat hypothalamus aqueous extract ^1^. SgII itself has been described previously in secretory granules in human astrocytes ^28^ and in mouse cerebellar brush cells ^29^, as well as in rat lateral hypothalamic neurons ^30, 31^ and endocrine cells of the small intestine ^32^. The tissue distribution of SgII, however, does not match that of the G530 binding site (as reported here and discussed previously ^1^). So G530 does not see full-length SgII. The G530 immunopurification campaign did not bring forth SgII-70 as such, but a larger protein identified by the MS software as Q8CGL8, a splice variant of rSgII ^1^. This item has a single non-C terminal NNI that the present work suggests would not be seen by the antibody. It can, however, be surmised that such an NNI might be seen in the presence of other relevant co-located non-contiguous epitope residues, notably F. This is perhaps why FNNA is preabsorptive. Western blots with G530 on sheep serum and rat PC12 conditioned medium visualized single bands at ~7+ kDa ^1^ These monobands related to extracellular secreted entities. In contrast, rat hypothalamus yielded three or more close bands around 7+ kDa, whether the aqueous extract was subjected to anion exchange chromatography or purified using a G530 affinity column ^1^. Staining intensity increased down the gels, with all bands preabsorbed by EPL001. The hypothalamic bands represent intracellular forms. They could be intermediates in the processing of SgII-70 towards secretion. This suggests that a non-C-terminal NNI becomes C-terminal, with the IHC exhibiting pre-SgII-70 as well as SgII-70. In this model, G530 is monoepitopic in both senses, towards the synthetic antigen and the endogenous antigen, but in the latter case there is more than one (appropriately folded) SgII-related form.

G530 labels features within the walls of the cerebrovasculature, labelling which is likely to represent at least in part the smooth muscle cell layer. This interpretation is strongly supported by the association of this labelling with that for LRP1, a marker for smooth muscle cells ^33^. No association has been reported between SgII and vascular smooth muscle cells, although secretoneurin (SN), a 33mer peptide derived from the proteolytic processing of SgII has angiogenic properties ^34^. The SN sequence does not contain NNI and has no overlap with that of EPL001. Another SgII peptide is EM66 ^35^. This 66mer does possess SgII’s NNI but the sequence of EM66 otherwise does not resemble that of EPL001 and includes no F. If a peptide, possibly with a C-terminal NNI, is processed from SgII then it must be derived via a different proteolytic pathway than SN or EM66.

Immunostaining was paradoxically enhanced by ANNI ( Figure 5 and Table 1, where ANNI is recorded as the NNI sequence in hSgII and sSgII). Binding of this tetramer to tissue can be suspected, via its alanine N terminus, providing additional NNI epitopes for the antibody to bind. The 14mer peptide EPL143 was preabsorptive ( Table 1). In this case a culminating NNI is preceded by K, showing that G530 may be able to recognize any C-terminal NNI, though the F tetramer data indicate that this is not the endogenous epitope in full. Molecular modelling upheld the immunosorbent trimer NNI as a passable representation in space of EPL001’s _12_NNI _14_, although the likeness narrowly escaped statistical significance. Referring to three peptides in particular, the spatial resemblance in each case to EPL001’s _11_FNNI _14_ is FNNI = MKPVFNNI (both significantly associated) > KEFNNI (NS). In contrast, the preabsorption power ranking is FNNI > KEFNNI > MKPVFNNI. The activity of KEFNNI supports the relevance of KE to the endogenous epitope, in addition to FNNI. (The relative weakness and variability of KEFNNI as a preabsorptive agent and the divergent dimensions of the hexamer from the parent peptide made alanine substitution of the hexamer an unpromising option.) MKPVFNNI, with lower immunosorbence, lacks an E. This indicates that E is a key component in the endogenous epitope, especially as the K-F gaps are similar in **K** E **F** NNI and M **K** PV **F** NNI.

Ovine SgII-9 is MLKTGEKPV (see *Introduction*), while human SgII-9 has one difference, involving a dissimilar type of amino acid: MLKTGEKP **N**. As IHC staining is seen in tissue sections from both species ^1^, the V in sSgII-9 and hence in EPL001’s C-terminal section ( _7_K **V** KEFNNI _14_) is arguably irrelevant to the epitope. Side-chain interatomic distances ( Figure 6: a–b and a–c) between K and E in sSgII-9 (ML **K** TG **E** KPV) are strikingly smaller, at ~3 Å, than those relating to the KE in EPL001 (MKPLTGKV **KE** FNNI), but those of EK in sSgII-9 (MLKTG **EK** PV), at a little under 12 Å, are similar to those of EPL001’s _9_KE _10_. Leaving aside any contribution to the epitope of nearby peptide backbone atoms and potential reverse-sequence steric differences, this first-order fit supports the deductions that the synthetic epitope of the G530 antibody is EPL001’s _9_ **KEFNNI**_14_ ( Figure 6, LYS 9 – ILE 14) and that the endogenous epitope is **KE·F·NNI**. This latter refines an earlier prediction ^1^ of K·E·F·NNI. By species, the SgII-related epitopes are proposed to be: sheep (W5QEU8), _373_ **KE**_372_· _?_ **F** · _236_ **NNI**_238_; human (P13521), _374_ **KE**_373_· _?_ **F** · _238_ **NNI**_240_; rat (P10362), _376_ **KE**_375_· _?_ **F** · _239_ **NNI**_241_; and mouse ( Q03517), _376_ **KE**_375_· _?_ **F** · _239_ **NNI**_241_.

The foregoing is consistent with there being a peptide derivative of SgII of ~70 residues that N-terminates in MLKTGEKPV/N and C-terminates in NNI, with these motifs sufficiently close together in space to be seen by an antibody. This is a piquant deduction because of residue numbering, which in sSgII is as follows: _367_MLKTGEKPV _375_ and _236_NNI _238_. Reverse peptide splicing is implied by the epitope mapping and the earlier NNI-omics or splicing from separate SgII molecules. Peptide splicing has been reported for another granin protein, chromogranin A, in an SgII-relevant intracellular locus, the secretory vesicle ^36^. Direct evidence for such splicing in relation to SgII will be sought next.

The immunosorbent power of EPL001 – against which peptide the G530 antibody was of course raised – overtops that of any of its C-terminal components in isolated form. The full 14mer alone seems to present the relevant residues in an appropriate consecutive approximation of a mixed endogenous epitope, for full binding. The relationship between the bioinformatically obscure EPL001 sequence and the proposed SgII-related endogenous antigen is in fact a circular conundrum: how can an endogenous protein be encoded in a synthetic peptide in such a way that an antibody to the synthetic peptide can get back to the endogenous protein? A speculative solution to this is as follows: faced with sSgII-70 the Edman machine did not provide a faithful N-terminal sequence (except for the initial methionine). Instead, it read available superficial residues, of the sort recognized indeed by antibodies. EPL001 thus represents epitope mapping by aberrant Edman sequencing. Hence an anti-EPL001 antibody recognizes sSgII-70. The reason that Edman sequencing was befuddled is deduced to be sSgII-70’s structure (relating perhaps to sorting domain chemistry), which lends itself to depolymerisation in the machine’s analytical chamber, a subject for further work.

EPL001 encodes residues in space, in the view just articulated, rather than being a faithful rendition of an endogenous sequence. Yet sequence alignments are not entirely lacking. There is a three-way doubleton match involving the homologue of SgII’s second sorting domain within the lead candidate fly antigen, the second sorting domain of SgII itself and EPL001: _1048_ **M**xxxxx **K**_1054_ (Q9W2X8), _367_ **M**xxxxx **K**_373_ (sSgII) and _1_ **M**xxxxx **K**_7_ (EPL001). The likelihood of this three-way cross-phylum match occurring by chance, with sorting-domain positionality, is <1 in 380,000 (see *Underlying data* ^37^).

Probing, via IHC preabsorption, an endogenous epitope that might be non-contiguous using a panel of short synthetic peptides, while requiring careful interpretation and a guiding hypothesis, has proved productive. A key insight is that antibody binding can be blocked with less than a full complement of epitope residues. Within the EPL001 14mer peptide MKPLTGKVKEFNNI, the epitope of the anti-EPL001 G530 antibody is evidently _9_ **KEFNNI**_14_. This must be so, as the endogenous epitope is deemed to be **KE·F·NNI**, a mixed contiguous and non-contiguous antibody binding site, as predicted by the hypothesis of antigen relatedness to SgII. The present data are thus consistent with the hypothesis that the anti-EPL001 antibody binds to an SgII related epitope. The postulated SgII-70 evidently N-terminates in MLKTGEKPV/N and C-terminates in NNI, crucial new considerations arising from the present study. EPL001 has arguably been decoded as SgII derived, ending an impasse. The next desideratum, en route to hormone substantiation, is SgII-70’s amino acid sequence in full.

## Data Availability

Figshare: Probability that a five residue sequence occurs in a protein by chance. https://doi.org/10.6084/m9.figshare.10265855.v1 ^7^. This project contains a statistical analysis of the likelihood of a five residue amino-acid motif occurring by chance. Figshare: Immunohistochemical labelling within the walls of blood vessels in the human cingulate cortex (BA24) with antibody G530. https://doi.org/10.6084/m9.figshare.9885536.v1 ^11^. This project contains raw images from Figure 1. Figshare: Human visual cortex labelled with G530 antibody or a commercial antibody to LRP1. https://doi.org/10.6084/m9.figshare.9879719.v1 ^12^. This project contains raw images from Figure 2. Figshare: Immunohistochemical labelling by antibody G530: blocking with cognate peptide. https://doi.org/10.6084/m9.figshare.9879734.v1 ^13^. This project contains raw images form Figure 3 Figshare: Immunohistochemical labelling of mouse small intestine by antibody G530. https://doi.org/10.6084/m9.figshare.9879773.v1 ^14^. This project contains raw images form Figure 4. Figshare: Immunohistochemistry with antibody G530 - tittering with tetramers. https://doi.org/10.6084/m9.figshare.9884996 ^15^. This project contains raw images form Figure 5. Figshare: Immunohistochemical labelling of mouse small intestine and rat brain by antibody G530 - Table data. https://doi.org/10.6084/m9.figshare.9884363.v1 ^16^. This project and the next two contain raw images behind Table 1. Figshare: Immunohistochemical labelling of mouse small intestine by antibody G530 - competition by tripeptides. https://doi.org/10.6084/m9.figshare.9884447.v1 ^17^. Figshare: Immunohistochemical labelling of mouse small intestine by antibody G530 - competition by tetramers. https://doi.org/10.6084/m9.figshare.9884534.v1 ^18^. Figshare: Immunohistochemical labelling of blood vessels within human visual cortex (BA17) and in mouse small intestine by antibody G530 - competition by KEFNNI and MKPVFNNI. https://doi.org/10.6084/m9.figshare.9884624.v1 ^19^. This project contains raw data discussed in the Results section. Newton, Russell (2019): Interatomic Distances Chi-Squared Test. figshare. Dataset. https://doi.org/10.6084/m9.figshare.9913040.v1 ^24^. This project contains inter-atomic distances for peptide sequences assessed in the study. Figshare: Ovis aries NNI-ome. figshare. Dataset. https://doi.org/10.6084/m9.figshare.10298204 ^26^ This project describes a bioinformatic analysis of proteins within the ovine predicted proteome containing the amino-acid motif NNI Figshare: The probability of MxxxxxK occurring by chance in three amino acid sequences. figshare. Dataset. https://doi.org/10.6084/m9.figshare.10298132 ^37^ This project contains a statistical analysis of the likelihood of three residue amino-acid motif occurring by chance. Data are available under the terms of the Creative Commons Attribution 4.0 International license (CC-BY 4.0).
