# Supplementary material for: Epitope mapping of an uncertain endogenous antigen implies secretogranin II peptide splicing
Source: F1000Res. 2019 Dec 5;8:1732. Originally published 2019 Oct 9. [Version 2] doi: 10.12688/f1000research.20633.2 (PMC7194351; doi:10.12688/f1000research.20633.2)
Supplement: Peer review history

# Peer review: Reviewer response for version 2

**DOI:** 10.5256/f1000research.23391.r58706

**Published:** 2020-04-28

**Authors:** Alice Sijts

**Affiliations:** 1 Department of Infectious Diseases and Immunology, Faculty of Veterinary Medicine, Utrecht University, Utrecht, The Netherlands

This is a somewhat unusual manuscript presenting many thoughts on how to identify an epitope, recognized by an antiserum that was raised against a synthetic peptide, identified by prior Edman sequencing.

 The authors decide to perform IHC on different tissues. The encouraging result is that they find that their antiserum stains these tissues, and that staining is blocked by smaller peptides, containing the C-terminal residues of the sequence against which the antiserum was raised. Based on these data, molecular modeling and bioinformatics analysis, the authors conclude that their antiserum may bind a 'secretogranin II-related' epitope. Since the epitope sequences are not encoded in linear SgII derivatives but all amino acid residues are present, the authors propose that the epitope, recognized by their antiserum, must be generated by peptide splicing.

 Overall, in its current form, I find this manuscript a bit too speculative - there is no direct evidence that the antiserum indeed recognizes an SgII-like protein-derived sequence that is formed by peptide splicing. My suggestion would be that the authors introduce the antigen candidates they describe into cell lines that don't stain with the antiserum themselves, and then perform IHC to identify the antigen recognized. They may then mutate this antigen, to verify whether the distant protein parts they suspect indeed are part of the epitope recognized. If so, more biochemical experiments (which do not necessarily have to be part of this manuscript) may be performed to confirm the role of peptide splicing in the generation of the current epitope.

Is the work clearly and accurately presented and does it cite the current literature?

Yes

If applicable, is the statistical analysis and its interpretation appropriate?

I cannot comment. A qualified statistician is required.

Are all the source data underlying the results available to ensure full reproducibility?

Yes

Is the study design appropriate and is the work technically sound?

Partly

Are the conclusions drawn adequately supported by the results?

Yes

Are sufficient details of methods and analysis provided to allow replication by others?

Yes

Reviewer Expertise:

NA

I confirm that I have read this submission and believe that I have an appropriate level of expertise to confirm that it is of an acceptable scientific standard, however I have significant reservations, as outlined above.

# Response

**Published:** 2020-04-30

**Authors:** D Howlett

**Affiliations:** Dr, UK

The authors are most grateful to Dr Alice Sijts for her thoughtful and interesting input – and for the contributions of the other reviewers too.  Where we are at the moment is that we think our antibody is seeing a noncontiguous epitope in proteolytic products of secretogranin II, among which is a secreted item, SgII-70.  Echoing the idea of Dr Sijts, finding or making a factor factory is a priority.  The intention is that our next paper, investigating bioactive peptides, will sport a suggested full primary sequence of ‘Sig-Two-Seventy’.

# Peer review: Reviewer response for version 2

**DOI:** 10.5256/f1000research.23391.r57504

**Published:** 2020-02-24

**Authors:** Steven D. Shnyder

**Affiliations:** 1 Institute of Cancer Therapeutics, University of Bradford, Bradford, UK

The authors have answered my concerns with their revisions and I think they are appropriate. Therefore I am now willing to approve the submission for indexing.

Is the work clearly and accurately presented and does it cite the current literature?

Yes

If applicable, is the statistical analysis and its interpretation appropriate?

I cannot comment. A qualified statistician is required.

Are all the source data underlying the results available to ensure full reproducibility?

Yes

Is the study design appropriate and is the work technically sound?

Partly

Are the conclusions drawn adequately supported by the results?

Yes

Are sufficient details of methods and analysis provided to allow replication by others?

Yes

Reviewer Expertise:

NA

I confirm that I have read this submission and believe that I have an appropriate level of expertise to confirm that it is of an acceptable scientific standard.

# Peer review: Reviewer response for version 1

**DOI:** 10.5256/f1000research.22692.r54975

**Published:** 2019-10-24

**Authors:** Steven D. Shnyder

**Affiliations:** 1 Institute of Cancer Therapeutics, University of Bradford, Bradford, UK

This study describes an attempt to clarify which epitope an antibody raised to a candidate peptide EPL001, G530, binds to.

 A rather crude methodology of using IHC with preabsorption of G530 with tripeptides was applied. One would normally expect a couple of techniques to be used to confirm the sequence, such as using a more sophisticated and definitive analytical method such as using MS, whereas in this study they have relied on the presence or absence of immunolabelling  on tissue sections, which is rather more subjective. The findings would have been more convincing if backed up using a more sophisticated analytical technique.

 As it stands I think the study would need to provide further experimental evidence to be worthy of indexing, such as including experiments where functional studies looking at SgII binding/blocking are carried out with G530 along with a known SgII antibody.

Is the work clearly and accurately presented and does it cite the current literature?

Yes

If applicable, is the statistical analysis and its interpretation appropriate?

I cannot comment. A qualified statistician is required.

Are all the source data underlying the results available to ensure full reproducibility?

Yes

Is the study design appropriate and is the work technically sound?

Partly

Are the conclusions drawn adequately supported by the results?

Yes

Are sufficient details of methods and analysis provided to allow replication by others?

Yes

Reviewer Expertise:

Tumour cell biology, proteomics, preclinical cancer pharmacology

I confirm that I have read this submission and believe that I have an appropriate level of expertise to confirm that it is of an acceptable scientific standard, however I have significant reservations, as outlined above.

# Response

**Published:** 2019-11-14

**Authors:** D Howlett

**Affiliations:** Dr, UK

We thank Dr Shnyder for his input.

The factor under investigation is refractory to MS, a constraint now highlighted in the revised version of the paper.

A research impasse called for highly unusual measures: the deployment of epitope mapping in a factor hunt (as spelt out in a new first sentence to *Introduction*), the use of IHC for epitope mapping (with automated image analysis to eliminate subjectivity), the adoption of rareified bioinformatics (newly uprated, see *Discussion* in particular), bespoke statistics (also uprated) and molecular modelling.

Confirming for Dr Shnyder, a known antibody to SgII was used in the study as a comparator and found to yield a different staining pattern to that of G530. To clarify the situation this sentence has been added to *Discussion*: ‘So, G530 does not see full-length SgII.’ 

The paper describes novelty under duress. The pursuit of an elusive factor goes on.

# Peer review: Reviewer response for version 1

**DOI:** 10.5256/f1000research.22692.r54922

**Published:** 2019-10-23

**Authors:** Michael O. Glocker

**Affiliations:** 1 Proteome Center Rostock, Medical Faculty and Natural Science Faculty, University of Rostock, Rostock, Germany

In the manuscript by Howlett *et al.* the authors report on their findings and thoughts on two main topics: (i) attempts to identify the endogenous antigen protein(s) which is (are) decorated by a polyclonal antibody (pAb), named G530, and through which are visualized e.g. certain blood vessel structures in tissues from different origin by IHC, and (ii) investigations targeted on defining the dominating epitope structure, starting with the amino acid sequence “MKPLTGKVKEFNNI”, named EPL001, which was taken to raise G530 and for which IHC is used as readout.

 The authors' research subjects are of high interest for the scientific community and stand in context with developing and applying state-of-the-art methods for elucidating antibody epitopes which is a necessity to understand scope and limitations of the applicability of antibodies as sophisticated research tools, as diagnostic tools, and so on.

 The authors' work underlying hypothesis seems to be: “Once the antibody’s target structure (which in effect is the antigen’s epitope structure) has been identified, the “endogenous” antigen(s) is (are) identified as well”.

 While this hypothesis seems logical and following it straight forward for successful antigen identification, the authors face the problem that the amino acid sequence stretch “MKPLTGKVKEFNNI” - which previously had been derived from Edman sequencing - cannot be matched to any known protein sequence of any of the species whose tissues had been investigated by IHC. Unfortunately, neither in this nor in the authors’ previous manuscript on the subject was the Edman sequencing result confirmed (see ref. 1 of Howlett *et al.* ^1^). Instead the authors admit indirectly that a rather poorly defined protein source had been applied for Edman sequencing (quote: *“…but scant amino acid sequence data could be obtained before the target molecule was lost to view.”*).

 In an attempt to overcome this shortcoming the authors speculate that the experimentally determined amino acid sequence “MKPLTGKVKEFNNI” might originate from a protein’s partial peptide which was produced by peptide splicing. The idea to assume peptide splicing as a cause of the determined amino acid sequence is driven by (rather weak) amino acid sequence similarities which seemed to point to SgII (or SgII-70) as a potential source of the amino acid sequence in question. Unfortunately, the authors do not provide evidence that peptide splicing should occur with SgII as substrate within the tissues which had been investigated by IHC.

 SgII was taken into consideration by the authors because this protein had been listed as potentially identified by IP followed by LC-MS analysis, as is stated. The authors report that in this particular case SgII identification was based on a single peptide match - out of a protein that in case of coming from *Drosophila* contains 1220 amino acids - when setting an FDR of 5%. Following suggested standards (see Carr *et al*. 2004; Mol. Cell. Proteom. 3, 531-533, 2004 ^2^) this identification result would rather be considered questionable. Of even more importance, finding a potential target protein by IP followed by LC-MS cannot replace precise characterization of an antigen’s total amino acid sequence prior to performing epitope mapping experiments. For determining an unknown amino acid sequence on the protein level, see e.g. Yefremova *et al*. J. Am. Soc. Mass Spectrom. (2015) 26:482-492 ^3^.

 Next, instead of repeating and/or improving antigen identification upon IP (or by other means) and despite not having unequivocally characterized the assumed antigen’s amino acid sequence, the authors had raised a polyclonal antibody, G530, against a synthetic peptide, named EPL001, which comprises the amino acid sequence “MKPLTGKVKEFNNI”. The authors show that (i) G530 recognizes certain blood vessel structures in tissues from different origin by IHC and (ii) G530-dependent IHC staining can be abolished by blocking G530 upon pre-incubation with EPL001.

 Encouraged by the antibody-related IHC staining pattern, the authors herewith justify their epitope mapping experiments which are described in this manuscript, despite the fact that their first try failed to identify the “endogenous” antigen by deducing its identity from its assumed epitope amino acid sequence “MKPLTGKVKEFNNI”.

 Epitope mapping with IHC as readout, as conducted in this manuscript, looks like an interesting alternative to other epitope mapping methods and starts with subsequently exposing the antibody of interest, in this case G530, to various peptides which do or do not show binding to the antibody. In this study peptide EPL001 and some derivatives therefrom were applied for pre-incubating G530 prior to conducting IHC staining experiments. However, one has to keep in mind that lack of IHC staining of the investigated tissue sections - which stands for saturation of the antibody’s paratope by peptide binding - is at best an indirect manner of epitope mapping and without appropriate controls lacks proof that loss of IHC staining is not caused by unrelated means, such as addition of detergents, pH change, etc. Unfortunately, the manuscript’s Experimental section does not provide enough information to estimate possible influence of such potential confounding factors. The authors are asked to provide more experimental details (see recommendations in the article guidelines: “Methods sections should provide sufficient details of the materials and methods used so that the work can be repeated by others.”). Also to be considered, binding of the peptide(s) under study to the antibody of interest is not shown directly by this method.

 Nevertheless, the authors performed the respective blocking experiments with various peptides, which are summarized in table 1 of this manuscript, and report that there are shorter partial peptide structures - with resemblances to EPL001 - which render negative IHC staining, hence block G530. From these results a “motif” of six amino acid residues (“KEFNNI”) is deduced by the authors as being necessary for binding G530 with both, the EPL001 peptide and the as of yet still unknown “endogenous” antigen. While the authors’ reasoning can be accepted for EPL001 and its shorter peptide derivatives, demanding that the “KEFNNI” motif must be present on the “endogenous” antigen of G530 is not automatically warranted.

 Moreover, one has to consider that the “KEFNNI” motif is precisely part of, but shorter than, the EPL001 peptide amino acid sequence and, therefore, adds no new information beyond what had been shown by dot blot experiments (contained in ref. 1, Figure 2). Consequently, the authors see themselves forced to narrow their base of their hypothesis on an even shorter piece of amino acid sequence as compared to that of EPL001, their first try with searching for the “endogenous” antigen using an amino acid sequence motif. In other words, the authors loosen stringency for data base search to find the mutual antigen and (as might have been expected) fail again in their attempt to convincingly identify the “endogenous” antigen of G530 by applying their “epitope amino acid sequence-based” strategy with focus on SgII as the potential “endogenous” target.

 In their attempts to provide more evidence on their reasoning the authors include results from molecular modelling approaches by which they intend to substantiate their assumptions about SgII being the “endogenous” antigen and to describe molecular structural features of EPL001 which might be required for antibody binding. Yet, these *in-silico* investigations remain theoretical and descriptive, hence, they ultimately stay inconclusive and are not convincing with respect to now “nailing” SgII as the “endogenous” antigen.

 Intriguingly, throughout this manuscript the authors apply methods whose data are to be interpreted rather indirectly in order to prove or falsify their hypothesis instead of using methods whose data provide results which can be directly interpreted to come to unequivocal conclusions. In other words, the authors try to compensate lacking experimental evidence with unproven theories. One is missing experiments which (i) deliver direct evidence about the nature of the epitope’s amino acid sequence(s) and (ii) allow determining the identity of the “endogenous” antigen. These circumstances are addressed by the authors in the discussion and outlook of this manuscript but their respective statements remain sketchy.

 More precise outlines about how the authors plan to continue with their attempts to experimentally determine the “endogenous” antigen of G530 ought to be added to this paragraph of the manuscript. The authors could mention that despite their first unsuccessful attempts with “aqueous” protein extracts it might seem more promising to retrieve the full length antigen protein, e.g. by immunoprecipitation, with protein extracts which also contain less soluble proteins (see e.g. DeCaprio and Kohl, Cold Spring Harb Protoc 2017 doi: 10.1101/pdb.prot098566 ^4^). They could mention that they intended to perform an in-depth characterization of the pulled-down and confirmed antigen protein, e.g. by mass spectrometric methods (see e.g. Yefremova *et al*. J. Am. Soc. Mass Spectrom. (2015) 26:482-492 ^3^). With respect to the epitope mapping and antibody recognition motif search, the authors could point to next apply methods which are capable to directly show binding to antibodies of peptides with varying amino acid sequences. A mass spectrometry-based method which is capable to do this is named “ITEM-THREE” and has recently been published by us (see Danquah *et al*. Mol. Cell. Proteom. (2019) 18:1543-1555 ^5^).

**Minor comments:**

 The term “primary sequence” ought to be deleted from the manuscript and replaced by either “amino acid sequence” or “primary structure”, depending on what of the two is to be described.

 The M+M section needs more precise descriptions so that the “storage conditions” of the peptides can be understood. The pre-incubation experiments need to be described in more detail. Buffer compositions, protein and peptide concentrations, and pH need to be given.

 How was the antiSGII antibody performance tested? Please add details. Without knowing whether the antibody is in fact capable of binding to SgII it is difficult to estimate the mentioned IHC results.

Is the work clearly and accurately presented and does it cite the current literature?

Yes

If applicable, is the statistical analysis and its interpretation appropriate?

Not applicable

Are all the source data underlying the results available to ensure full reproducibility?

Partly

Is the study design appropriate and is the work technically sound?

Partly

Are the conclusions drawn adequately supported by the results?

Partly

Are sufficient details of methods and analysis provided to allow replication by others?

Partly

Reviewer Expertise:

Mass spectrometry, proteome research, protein structure and function analysis

I confirm that I have read this submission and believe that I have an appropriate level of expertise to confirm that it is of an acceptable scientific standard, however I have significant reservations, as outlined above.

## References

[1] : Mysterious inhibitory cell regulator investigated and found likely to be secretogranin II related. *PeerJ* .2017;5: 10.7717/peerj.3833 e3833 10.7717/peerj.3833 29043108PMC5642266

[2] : The need for guidelines in publication of peptide and protein identification data: Working Group on Publication Guidelines for Peptide and Protein Identification Data. *Mol Cell Proteomics* .2004;3(6) : 10.1074/mcp.T400006-MCP200 531-3 10.1074/mcp.T400006-MCP200 15075378

[3] : Intact Transition Epitope Mapping (ITEM). *J Am Soc Mass Spectrom* .28(8) : 10.1007/s13361-017-1654-7 1612-1622 10.1007/s13361-017-1654-7 28616748

[4] : Detergent Lysis of Animal Tissues for Immunoprecipitation. *Cold Spring Harb Protoc* .2017;2017(12) : 10.1101/pdb.prot098566 pdb.prot098566 10.1101/pdb.prot098566 29196600

[5] : Intact Transition Epitope Mapping - Targeted High-Energy Rupture of Extracted Epitopes (ITEM-THREE). *Mol Cell Proteomics* .2019;18(8) : 10.1074/mcp.RA119.001429 1543-1555 10.1074/mcp.RA119.001429 31147491PMC6683010

# Response

**Published:** 2019-11-23

**Authors:** D Howlett

**Affiliations:** Dr, UK

We are honoured to receive Professor Glocker’s extensive analysis.

1. **EPL001.** The EPL001 sequence has been a deep source of perplexity. As described in *Introduction* it was seen once unambiguously during physicochemical purification and three times less fully, with appropriate bioactivity and MALDI MS correlates. Synthesized as a 14mer peptide EPL001 surprised by displaying anti-organotrophic and reproductive effects, as described in *Introduction* and *Discussion*, in line with the hypothesis underlying the entire project, of the existence of a tissue-mass reducing reproductively related hormone. Anti-EPL001 antibodies provided images of neuroendocrine relevance and immunoneutralised relevant bioactivities. Even though it is bioinformatically obscure, EPL001 is impossible to ignore, in our view: it is telling us something.

2. **Potential use of MS in epitope mapping. **The project experience with MS has been productive overall but problematic. We chose IHC for epitope mapping because we felt there was a reasonable probability of success, even though IHC is not a recognised route to defining antibody binding. The probability of success in MS-enabled epitope mapping, which in the case Professor Glocker’s outstanding method features a proteolytic enzyme, is more conjectural, as the history of the use of MS in the present project indicates, particularly in regard to the curious use of formalin fixation. The sheep plasma fraction from which the Edman EPL001 sequence was obtained yielded a peak in MALDI-TOF MS at *m/z* 7583.64 (Fig. 2 in Hart JE, 2013. Proteinaceous compounds: US patent 8367801, also EP 2234632, 2014; available at https://www.google.com/patents/US8367801?dq=Proteinaceous+compounds.&cl=en). Small peaks like this could be obtained in MALDI by hitting samples hard with the laser. Electrospray ionisation yielded nothing. The use of proteolysis with multiple MS modalities (see new section in *Introduction*) was similarly fruitless. Orbitrap LC-MS with trypsinisation eventually took the project to ‘SgII relatedness’, which evaluation cohered with the evidence from IHC, purification, westerns and so on, including a fruit fly candidate antigen Q9W2X8 that itself unexpectedly turned out to be granin-like. (MS IDs were on the basis of sole tryptic peptides, granted, but the mammalian and fruit fly proteins each had homologues of the other’s identifier, as newly described in an extended section of *Introduction*, involving new statistics and bioinformatics.) This apparent SgII advance was on the basis of formalin crosslinked feedstock in an immunoprecipitation campaign featuring aqueous extract of rat hypothalamus. Frozen unfixed material provided no credible hits. Formalin fixation with antigen retrieval had been learnt from IHC. We have found no precedents for the use of formalin in factor hunt purification and MS. The need for crosslinking presumably speaks of factor lability. Notably, though, the sought-for 70mer was not found. Instead a larger SgII entity was identified, putatively a processed intermediate. More important than further epitope work now is a full amino acid sequence.

3. **The connection with SgII. **The IHC images in reference 1 are granin-like. They could be taken straight out of a paper on one of the SgII derived peptides. The granin suspicion starts with IHC, not with purification MS or bioinformatics. Referring to bioinformatics, there are two kinds in the paper: the existing material is in *Introduction *and refers to the second sorting domain of SgII. The bioinformatics in *Discussion* has been subject to significant augmentation on the basis of ‘NNI-omics’. The NNI-ome is that part of the ovine proteome comprising proteins with at least one example of the motif NNI, the final triplet in EPL001 important in the epitope of the anti-EPL001 antibody G530. The sift reaches SgII as the lead candidate. In *Discussion *there is a new pen-ultimate paragraph. This in effect describes a sequence alignment between EPL001, sSgII and the fly protein Q9W2X8 as either meaningful or a remarkably freakish coincidence. In our view, the sought-for factor is very likely to be secretogranin II related.

Although an interim report following the 2017 paper (reference 1), the present account is proffered as a paper of record, which colleagues will need in efforts at confirmation or refutation. It might also be of interest to the epitope mapping community.

Professor Glocker is thanked for stimulating many improvements to our paper.
